# Intelligent effort: Involving citizens in planning for quality in nursing

**DOI:** 10.1002/nop2.1157

**Published:** 2021-12-18

**Authors:** Bethany Taylor, Steve Robertson, Tony Ryan

**Affiliations:** ^1^ Division of Nursing & Midwifery The University of Sheffield Sheffield UK; ^2^ Leeds Beckett University Leeds UK

On the global stage, nurses have been recognized by the World Health Organization (WHO) as being critical in the effort to achieve the Sustainable Development Goals (SDGs) through a range of contributions (World Health Organisation, 2020). The COVID‐19 crisis has reinforced this, highlighting just how crucial nurses are, not only in providing often under‐appreciated day‐to‐day care in a range of settings, but in flexibly and professionally responding to crises often at great personal cost (Catania et al., 2021). Other challenges remain. The outcomes of climate change, shifts in population demographics and migration patterns, civil unrest and the risk of further respiratory (and other) pandemics all create health risks that even excellent technological advances are struggling to keep up with. Within this context, nursing contributions need to be maximized in order to fully address these global health challenges.

Yet such contributions do not happen by chance. A range of core values such as honesty, integrity and human dignity, alongside proficiency in practical skills, are required when training and developing nurses to become competent and experienced care providers. The ability to critically reflect is also key to becoming a successful, autonomous practitioner who continues to innovate in order to provide high‐quality safe and effective care across a range of contexts. Furthermore, policy and service delivery interventions are needed that facilitate and fully release this potential by allowing these skilled, innovative, autonomous nurses to thrive.

The topic of this special issue arose when a team of researchers, funded through a Royal College of Nursing (RCN) grant, began to consider what the key issues are that facilitate or inhibit access to quality nursing care. Variations in care, and high profile cases of poor care that have led to safety breaches and increased mortality, have rightly placed this issue of quality care at the top of the UK nursing professions agenda, as is often the case around the world.

However, identifying and reaching agreement on what the key factors are that impact on safe and effective care is not easy. This special issue focuses on a development project that planned to bring together experts and members of the public, with the aim of delivering a Consensus statement focusing on nursing policy and practice. The first paper by Taylor et al. (2021) therefore outlines the Consensus processes that were undertaken to try to garner such agreement. The authors note how these processes involved combining literature and policy reviewing with seeking lay and expert opinion on emerging areas in the move towards Consensus. They further highlight the challenges of amending the more usual face‐to‐face Consensus methods to virtual methods in the context of COVID‐19 and, in so doing, illuminate key learning points for those planning future, virtual, Consensus events.

The ability to care for the whole person has long been said to be the essence, the very centre, of good nursing practice (Kitson, 1999; Leininger, 1991) and is a core theme in many countries regulatory nursing standards. However, debates about what constitutes person‐ and relationship‐centred care continue. The second paper by Ryan (2021) distils out common themes of these concepts across a broad range of literature and considers some of the models available before highlighting factors, at both individual and organizational level, that facilitate such care.

One of the major challenges to delivering high‐quality, safe and effective person‐centred care is the global shortage of nurses. As Catton (2020) notes, “The future resilience of healthcare services will depend on having sufficient numbers of nurses who are adequately resourced to face the coming challenges” (p.4). The third paper by Ball and Griffiths (2021) therefore provides an overview of the research evidence on nurse staffing levels in adult acute care and their relationship to health outcomes focusing mainly on the UK. The paper not only examines what might constitute safe staffing levels but also considers what evidence is available on skill mix in the delivery of nursing care and how current evidence has been applied in policy and practice. Closely linked to the issue of safe staffing levels is the amount of nursing care left undone, or “missed care.” The fourth paper by Willis and Brady (2021) picks up this key concern in contemporary nursing and provides evidence on the outcomes of missed care in relation to “mortality,” “adverse events” and “failure to maintain” (as an antecedent of adverse events). However, it also highlights the methodological difficulties involved in making direct causal links between missed care and increased mortality, adverse events or failure to maintain but does highlight missed nursing care as a global phenomenon.

The development of technically proficient, innovative and caring nurse practitioners mentioned earlier in this editorial requires ongoing learning beyond initial registration. The final paper in this special issue, by Jackson and Manley (2021), therefore considers the part that continuing professional development (CPD) can play in influencing the quality of care experienced by citizens and communities. The authors highlight the importance of capitalizing on the workplace as a learning resource to facilitate development, improvement, and knowledge translation. This, they say, requires skilled facilitators who can develop system‐wide cultures of learning that enable nurses to flourish. They summarize by suggesting a shift in emphasis from individuals and organizations to teams and systems built around facilitating the effective allocation of often scarce CPD resources to optimize benefit for patients, citizens and practitioners, thereby maximizing nurses contribution to person‐centred, safe and effective care.

This special issue brings together those expert positions on a range of important areas of nursing practice and places these in the context of existing evidence and policy. Importantly, and as part of the Consensus building process, the inclusion of the voice of members of the public has enabled the emergence of a set of priorities and standards for us all to work with. The full Consensus statement can be accessed here [https://sites.google.com/sheffield.ac.uk/sra/home/Consensus‐development]. This statement will, of course, not provide all of the solutions to the challenges facing nurses and the people that they care for. What it will do is introduce a further opportunity for debate, discussion and provides a legitimate marker with which we can hold decision makers to account. One thing that these special issue papers make abundantly clear, as John Ruskin the nineteenth‐century writer and philosopher said, is that “Quality is never an accident, it is always the result of intelligent effort.”
